# Preliminary analysis of immune responses in patients enrolled in a Phase II trial of cyclophosphamide with allogenic dribble vaccine alone (DPV-001) or with GM-CSF or imiquimod for adjuvant treatment of stage IIIa or IIIb NSCLC

**DOI:** 10.1186/2051-1426-3-S2-P435

**Published:** 2015-11-04

**Authors:** Rachel Sanborn, Brian Boulmay, Rui Li, Bradley Spieler, Kyle Happel, Christopher Paustian, Tarsem Mougdil, Zipei Feng, Christopher Dubay, Brenda Fisher, Yoshinobu Koguchi, Sandra Aung, Eileen Mederos, Carlo Bifulco, Michael McNamara, Keith S Bahjat, William Redmond, Augusto C Ochoa, Hong Ming Hu, Bernard Fox, Walter Urba, Traci Hilton

**Affiliations:** 1 Robert W. Franz Cancer Research Center, Earle A. Chiles Research Institute, Providence Cancer Center, Portland, OR, USA; 2 Stanley S. Scott Cancer Center, School of Medicine, LSUHSC, New Orleans, LA, New Orleans, LA, USA; 3 Providence Cancer Center, Portland, OR, USA; 4 Earle A. Chiles Research Institute, Providence Cancer Center, Portland, OR, USA; 5 Earle A. Chiles Research Institute, Portland, OR, USA; 6 Prometheus Laboratories Inc., San Diego, CA, USA; 7 UbiVac, Portland, OR, USA

## Background

Tumor-derived autophagosomes, DRibbles, are DC-targeted microvesicles containing more than 100 putative NSCLC antigens, many as potential altered-peptide ligands (APL), which could increase their immunogenicity. The microvesicles also contain at least 15 DAMPs with agonist activity for TLR 2, 3, 4, 7 and 9. In preclinical models DRibble immunotherapy provided significant cross-protection against 8 of 9 tumors and was effective in treating established tumors. We hypothesize that the efficacy of DRibbles' vaccination stems from their ability to present stabilized tumor-derived short-lived proteins (SLiPs) and defective ribosomal products (DRiPs) that are normally not processed and presented by professional antigen presenting cells (APCs). These SLiPs and DRiPs represent a potential pool of tumor antigens against which the host is not tolerant. The DPV-001 vaccine is made up of DRibbles produced from two cell lines, UbiLT3 and UbiLT6. The priming vaccination includes DRibbles from both cell lines, while subsequent administrations alternate between DRibbles derived from the two cell lines.

## Methods and results

Patients are vaccinated at 3-week intervals, alone or with assigned adjuvant (GM-CSF or imiquimod), for a total of 7 doses, and an option to continue vaccinating at 6 week intervals (without adjuvant). PBMCs and serum are collected at baseline and at each vaccination. Immune monitoring panels are run on peripheral whole blood to evaluate lymphocyte populations and their activation status. PBMCs from the baseline visit and week 12/13 are stimulated with DRibbles (vaccine and control) to measure vaccine-specific cytokine production. Patient serum from the baseline visit and week 12/13 is analyzed for antibody response to >9000 human proteins using ProtoArrays. Whole exome sequencing of tumors and normal tissue is performed when possible to evaluate antibody responses to mutations and altered peptide ligands. Nanostring and multispectral IHC are being used to evaluate tumor immune biomarkers when tissue is available.

## Conclusions

The DPV-001 vaccine provides a source of broad-spectrum relevant antigens. Preliminary analyses of patients receiving the DPV-001 vaccine show effects on T cells and B cells with increased antibody responses at 12 or 13 weeks.

Clinical Trial Identifier: NCT01909752, Support: R44 CA121612-02A1.

## Trial Registration

ClinicalTrials.gov identifier NCT01909752.

